# Spontaneous cervical epidural hematoma: Report of a case managed conservatively

**DOI:** 10.4103/0019-5413.41863

**Published:** 2008

**Authors:** Tariq Abdul Halim, Vishal Nigam, Vikas Tandon, HS Chhabra

**Affiliations:** Department of Orthopedics Surgery, Indian Spinal Injuries Centre, Sector C, Vasant Kunj, New Delhi - 110 070, India

**Keywords:** Cervical epidural hematoma, conservative management

## Abstract

Spontaneous spinal epidural hematoma is a rare cause of acute spinal cord compression. A 25-year-old male presented with a history of sudden onset of complete quadriplegia with sensory loss below the neck along with loss of bowel and bladder control. He had no history of any constitutional symptoms. He reported 10 days later. He was managed conservatively and after two weeks of intensive rehabilitation he had complete neural recovery.

The spontaneous recovery of neurological impairment is attributed to the spreading of the hematoma throughout the epidural space, thus decreasing the pressure with partial neural recovery.

Conservative treatment is a fair option in young patients who present late and show neurological improvement. The neurological status on presentation will guide the further approach to management.

## INTRODUCTION

Spontaneous spinal epidural hematoma is a rare cause of acute spinal cord compression. Clinically it can present through a wide neurological deficit ranging from simple cervical radiculopathy to complete quadriplegia depending on the severity and rapidity of compression. They have been reported in association with blood dyscrasias, coagulopathies, anti-coagulant treatment, infection, tumor, pregnancy and vascular malformations but the vast majority doesn't have a definite cause.^1^

The spinal epidural hematoma of cervical spine is usually spontaneous and of acute onset^2^ while hematomas occurring at lower levels of the spine have a more subacute or chronic course. Moreover, in the latter, the cause of the bleeding is more likely to be defined.^3^

Although the currently suggested approach is an urgent surgical decompression,^4^,^5^ conservative treatment is recommended when there is an objective improvement of the neurological status.^6^,^7^ The case reported here illustrates the neural recovery without surgical intervention.

## CASE HISTORY

**Figure 1:**
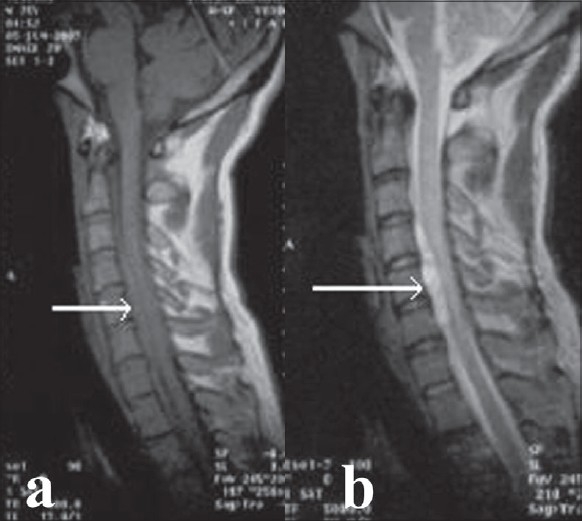
Mid sagittal T1 (a) and T2 (b) weighted MRI of cervical spine at onset of weakness shows epidural mass (solid arrow)

A 25-year-old male developed sudden onset of complete quadriplegia with sensory loss below neck along with loss of bowel and bladder control. There was no history of trauma, constitutional symptoms or any coagulopathy. Magnetic resonance imaging (MRI) done at the time of onset revealed epidural collection from C2-T1 with cord compression [Figure 1a,b]. He was initially managed conservatively.

Ten days later he reported to us; he had improvement in his motor power and partial recovery of sensations along with recovery of bladder sensations. Neurological assessment on admission revealed that he had a motor power of 4/5 on the right and 5/5 on the left side in all muscle groups of the upper limbs. In the lower limbs he had a power of 3/5 in the muscle groups of bilateral hips and knee and 4/5 in the muscle groups around his bilateral ankles. Sensations were impaired below C4. Perianal sensations and voluntary anal contraction were present. He had bladder sensations without control along with normal bowel sensations and control. His blood parameters along with coagulation profile were normal. A spinal artery Digital Substraction Angiography (DSA) was done to rule out any arterio-venous malformation.

**Figure 2:**
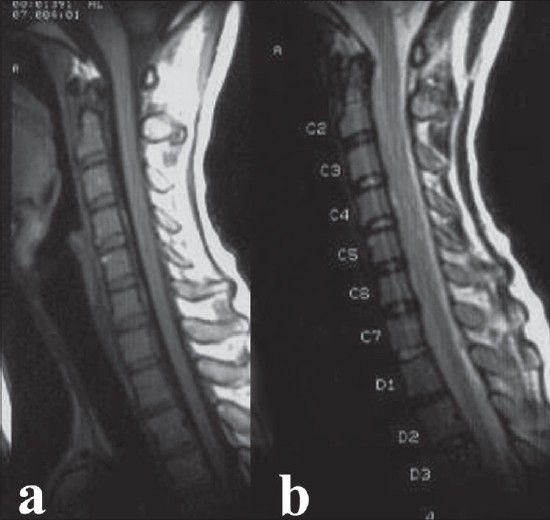
Mid sagittal T1 (a) & T2 (b) weighted MRI of cervical spine 11 days after first MRI shows complete resolution of hematoma

As the patient had shown significant neurological improvement in the 10 days after the onset of complete quadriplegia, he was managed conservatively at our center. Magnetic resonance imaging done after admission (11 days after first MRI) revealed resolution of hematoma [Figure 2a, b]. He was advised intensive rehabilitation, and had complete recovery of sensations, control of bladder and bowel along with complete motor recovery and the patient was walking without support at the time of discharge.

## DISCUSSION

Spontaneous spinal epidural hematomas are rare and relatively few cases have been described in the literature. Most reports are of a single case such as this one or are based on a small number of patients. The etiology is unknown, but it is generally accepted that most of these hematomas arise from rupture of the epidural venous plexus.^8^ However, it is still being debated whether the source of bleeding in acute spontaneous cervical hematomas is arterial or venous.

Bruyn and Bosma^1^ theorized that spontaneous spinal epidural hematomas occur by the following mechanism: there is local pooling within valve less, thin walled epidural veins and brief increases in intravenous pressure, because of increased intrathoracic and intraabdominal pressure, may lead to their rupture. This might account for those cases reported to have occurred in association with voiding, bending, turning in bed at night, straining at stool, sneezing and coitus. While this explanation may be valid for the subacute or chronic hematomas of the lumbar region, it is unsatisfactory to explain acute spontaneous cervical epidural hematomas (ASCEH). Venous pressure is low in epidural veins of the cervical region, even lower than the intrathecal pressure. In addition the rapidity with which spinal cord compression develops tends to favor arterial bleeding. Beaty and Winston^2^ postulated that the source of bleeding was the 'free' anastomotic arteries that run in the epidural space and connect with the radicular arteries. Since the majority of the ASCEH are located in the C6/7 region, a highly mobile segment of the spine, they believed that certain movements at this level might stretch the free arteries beyond their limits of tolerance, causing them to rupture.

The most common presentation is an acute onset of pain and radicular symptoms that mimic disc herniation. Depending on the severity and rapidity of bleed, there may be a rapid progression of neurological symptoms. There is no significant correlation with age, race or gender and there is disagreement in the literature as to the most common site in the spine.

The T1 weighted images of MRI has been shown to be the most valuable and the initial images show an epidural mass that is isointense to the cord and this is the most important feature when the patient is imaged within 48 h of the onset of the symptoms. In post-traumatic spinal epidural hematomas, however, the blood seen on T1 images appears hyperintense relative to the cord because of the effects of methemoglobin.^9^

Differential diagnosis is highly dependent on the appropriate clinical radiological correlation. The primary considerations include epidural abscess and spinal epidural lymphoma. The clinical and laboratory findings should help exclude infection and the imaging features are the most critical in excluding neoplasm.

The postulated hypothesis for the spontaneous recovery of neurological impairment is the spreading of the hematoma throughout the epidural space, thus decreasing the pressure.^10^,^11^ The prognosis appears to be related to the severity of the preoperative neurological deficit and many early reports suggest an early operation for rapid decompression as crucial.^12^ Lawton *et al.*, confirmed the relationship between neurological recovery, the timing of surgery and the preoperative neurological status.^13^ However, subsequent reports indicate that the course of the disease is relatively benign in many cases especially in young patients even with conservative treatment. Holtas *et al.*, concluded that age is probably one of the most important factors in determining outcome.^8^ In the literature, geographic isolation, initial inaccurate diagnosis, neurological improvement prior to presentation, high surgical risks and several other causes have been cited as reasons that have led to the opportunity for spontaneous recovery.

Conservative treatment is currently indicated in uncommon situations or when neurological symptoms improve before medical evaluation, as was the case who presented to us with significant neurological recovery after 10 days of onset of symptoms.

Hence we conclude that conservative treatment is a fair option in young patients who present late and show neurological improvement. Neurological status on presentation will guide the further approach to management.
